# Minimally invasive lateral plating for diaphyseal fractures with extension into the proximal humerus and its implications for the deltoid muscle and its distal insertion: functional analysis and MR-imaging

**DOI:** 10.1186/s12891-023-07004-6

**Published:** 2023-11-08

**Authors:** D Flury, C Metzler, S Rauch, M Schläppi, E Benninger, C Meier

**Affiliations:** 1 Division for Orthopaedics and Traumatology, Cantonal Hospital Winterthur, Brauerstrasse 15, Winterthur, 8401 Switzerland; 2 Department of Radiology and Nuclear Medicine, Canton Hospital Winterthur, Brauerstrasse 15, Winterthur, 8401 Switzerland

**Keywords:** Minimally invasive lateral plate osteosynthesis of the humerus, Distal deltoid insertion, Shoulder function, Abduction strength, Deltoid muscle, MR imaging

## Abstract

**Background:**

In minimally invasive lateral plate osteosynthesis of the humerus (MILPOH) the plate is introduced through a deltoid split proximally and advanced through the central portion of the deltoid insertion and between bone and brachial muscle to the distal aspect of the humerus. The fracture is then indirectly reduced and bridged by the plate. Whereas it has been shown that the strong anterior and posterior parts of the distal deltoid insertion remain intact with this maneuver, its impact on deltoid muscle strength and muscular morphology remains unclear. It was the aim of this study to evaluate deltoid muscle function and MR-morphology of the deltoid muscle and its distal insertion after MILPOH.

**Methods:**

Six patients (median age 63 years, range 52–69 years, f/m 5/1) who had undergone MILPOH for diaphyseal humeral fractures extending into the proximal metaphysis and head (AO 12B/C(i)) between 08/2017 and 08/2020 were included. Functional testing was performed for the injured and uninjured extremity including strength measurements for 30/60/90° shoulder abduction and flexion at least one year postoperatively. Constant-Murley-Score (CMS) including an age-and gender-adjusted version, were obtained and compared to the uninjured side. Oxford Shoulder Score (OSS) and the Disability of the Arm, Shoulder and Hand (DASH) questionnaire were acquired for the affected extremity. Quality of life was measured using the EQ visual analogue scale (EQ-5D-5 L VAS). MR imaging was performed for both shoulders accordingly at the time of follow-up to assess the integrity of the distal insertion, muscle mass and fatty degeneration of the deltoid muscle. Muscle mass was determined by measuring the area of the deltoid muscle on the axial MR image at the height of the center of the humeral head.

**Results:**

Median follow-up was 29 months (range 12–48 months). Median difference of abduction strength after MILPOH was + 13% for 30°, 0% for 60° and − 22% for 90°. For flexion, the difference to the uninjured side was measured 5% for 30°, -7% for 60° and − 12% for 90°. Median CMS was 75 (66–82) for the operated extremity compared to 82 (77–90) for the uninjured side. Age- and gender-adapted CMS was calculated 88 (79–99) vs. 96 (89–107). Median OSS was 47 (40–48). DASH was 26 (15–36). EQ-5D-5 L VAS ranged from 81 to 95 with a median of 90. The median difference of the deltoid muscle area on MRI was 2% (-21% to + 53%) compared to the uninjured side. No fatty degeneration of the deltoid muscle was observed. The weaker central part of the distal deltoid insertion was exclusively perforated by the plate, leaving the strong anterior and posterior parts of the insertion intact in all patients.

**Conclusions:**

MILPOH was associated with good functional and subjective outcome. Minor impairment of abduction strength was observed with increasing abduction angles. The reason for this impairment is unclear since MILPOH did not affect the structural quality of the deltoid muscle and the integrity of the strong anterior and posterior parts of its insertion remained intact.

**Trial registration:**

26/05/2023: ISRCTN51786146.

## Background

Minimally invasive plate osteosythesis (MIPO) of the humerus has gained increased popularity among surgeons in recent years. Subtle indirect reduction techniques are combined with percutaneous submuscular placement of locking plates to preserve the fracture hematoma and the remaining osseous blood supply for improved bone healing. Compared to open reduction and internal fixation (ORIF) lower rates of non-unions were reported with a generally favourable outcome for humeral shaft fractures [1]. MIPO may also associated with less non-unions, less re-interventions and improved shoulder function compared to intramedullary nailing of humeral shaft fractures [2]. Different techniques have been described such as anterior or lateral plate positions [3, 4]. Whereas anterior plates are not suitable for fractures extending proximally towards the humeral head, potential injury to the radial nerve is a concern for lateral plate positioning [5]. Helical plates have been used to avoid potential injury to both the distal deltoid insertion and the radial nerve [6]. In minimally invasive lateral plate osteosynthesis of the humerus (MILPOH), the distal deltoid insertion may be injured when the plate is pushed distally as shown in a previous cadaveric study [7]. In that study, only the weaker central parts of the insertion were perforated leaving the strong anterior and posterior parts of the distal deltoid insertion intact in all specimen. Whereas the risk of injury to the radial nerve for lateral plates has been investigated in several clinical studies, the functional implication of bluntly perforating the central parts of the distal deltoid insertion remains unclear. As the affected portions of the deltoid muscle correspond with the proximal insertion arising from the lateral edge of the acromion, distinct impairment of muscle function combined with muscle atrophy and fatty infiltration of these muscle portions maybe expected [8]. However, isolated testing of the deltoid function is not possible in a clinical setting since the deltoid muscle and the merging rotator cuff act synergistically for abduction and flexion of the shoulder. In a recent study, it was shown that the force distribution for the deltoid muscle increases in a linear manner from 0° to 120° of abduction and flexion, respectively with a higher overall contribution of the deltoid muscle to abduction strength than to flexion strength [9]. It was the aim of this study to evaluate the implications of MILPO on the integrity of the deltoid insertion, muscle morphology, and functional outcome as well. We hypothesized that the blunt perforation of the weaker central part of the distal deltoid insertion does not lead to a relevant decrease of deltoid muscle function due to the preserved integrity of the important anterior and posterior insertions. To our knowledge, this has never been studied before.

## Methods

### Patients

The charts of all patients with MILPOH for diaphyseal fractures of the humerus with extension into the proximal metaphysis, operated at our institution between 03/2017 and 08/2020 were reviewed. Only patients with normal function of the affected extremity and the contralateral side before trauma were eligible for this study. Subjects with previous shoulder or upper arm surgery on either side were excluded. The remaining candidates were contacted and informed about the study. Only volunteers > 18 years of age agreeing to undergo functional testing and MRI imaging of both shoulders and upper arm at least 12 months following surgery were included. The study protocol was approved by the local ethical committee and informed consent for the purpose of the study was obtained from all study participants. The study was registered in the ISRCTN Registry on 26/05/2023 (ISRCTN51786146). The authors had access to information that could identify individual participants during or after data collection. However, the two senior radiologists, who analysed the MR images, had no access to any clinical data concerning shoulder function of the participating patients.

### Surgical procedure and postoperative management

**Fig. 1:**
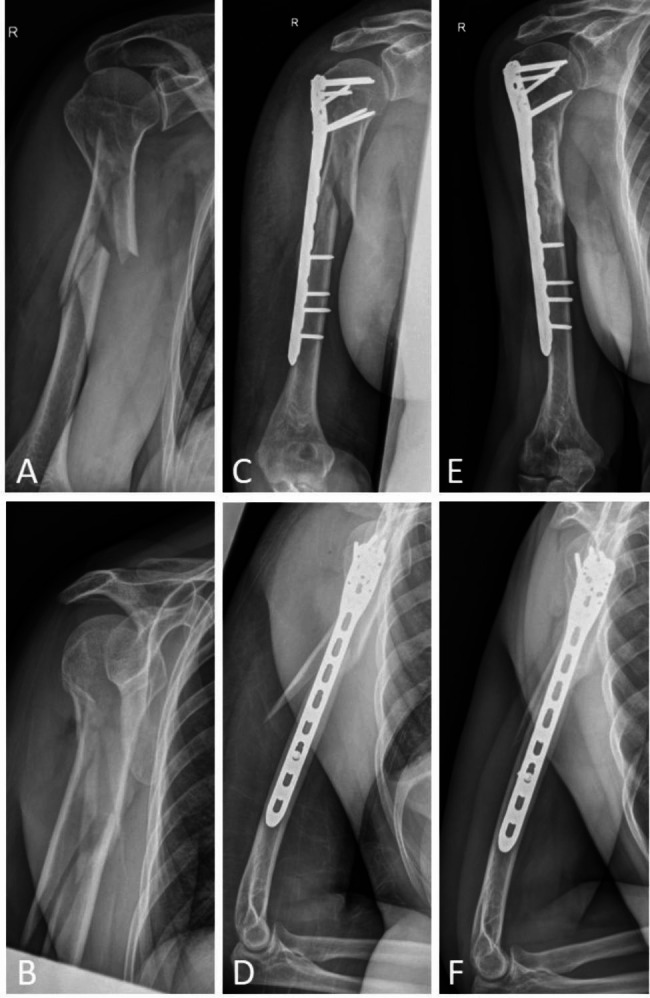
Case example of a 59-year old woman after a stair fall. **A/B**. The multifragmentary diaphyseal fracture with extension into the proximal metaphysis (AO 12C2(i)) was bridged with a minimally invasive lateral plate osteosynthesis the same day. **C/D**. Postoperative radiographs show good fracture alignment and plate position. **E/F**. At 12-month follow-up radiographs demonstrate bone healing

Patients were operated in a beach chair position. Depending on the anatomy and fracture extension, an appropriate long PHILOS™ plate (Proximal Humeral Internal Locking System, 8–10 holes, Depuys Synthes, Oberdorf, Switzerland) was used. Following an incision over the lateral aspect of the distal humerus distally to the fracture, the radial nerve was identified and protected. Through a deltoid split approach, the plate was introduced onto the periosteum of the greater tuberosity and slid distally. Once the resistance increased, the plate was bluntly pushed through the deltoid insertion and advanced underneath the brachial muscle to its final position. Indirect reduction manoeuvres were performed to align the fracture before definitive fixation. The surgical technique is described in detail elsewhere [5]. A case example is shown in Fig. 1.

Postoperatively, the arm was immobilized in a sling for 2 weeks. Active and passive physiotherapy without limitation of the allowed range of motion was initiated 2 days after surgery. Strengthening exercises were started between 6- and 12 weeks postoperatively. Neither neurotrophic medication nor electric stimulations were administered, pain control followed the WHO analgesic ladder.

### Functional measurements and patient-reported outcome measures (PROMS)

Strength measurements were performed for the injured arm at least 1 year after surgery between 12/07/2020 and 08/18/2021. The contralateral side was tested as well and served as control. Abduction strength with the elbow extended and the wrist pronated was measured in the coronal plane for 30°, 60°, and 90° of abduction. Flexion strength was measured accordingly in the sagittal plane for 30°, 60°, and 90° of flexion. These angles were set with a standard goniometer with the patient in a standing position. Since 120° of abduction or flexion was not reached by all our patients with their injured shoulder, the maximal angles to measure strength were set at 90°.

An electronic isometric strength dynamometer (IsoForceControl EVO2; Medical Device Solutions AG, Oberburg, Switzerland) was attached to a wall or table to make sure that the pulling force was always perpendicular to the chosen abduction or flexion of the arm. The loop of the dynamometer was placed at the wrist and peak strength was measured.

The Constant-Murley Score (CMS) was obtained for the injured and uninjured side [10]. The age- and gender-adjusted CMS was calculated according to Katolik et al. [11]. The patients completed the Oxford Shoulder Score (OSS) for the injured side, consisting of 12 multiple-choice questions with four possible answers for each question [12]. The questionnaire covers pain and daily life activities with a score ranging from 0 to 48 points. The higher the score the better the joint function. The Disabilities of the Arm, Shoulder, and Hand (DASH) outcome measure was obtained for the affected extremity as well. It consists of 30 questions, which cover daily life activities, specific symptoms and social or occupational limitations [13]. The measure ranges from 0 to 100 points. Zero points represent a normal unrestricted function of the upper extremities. The DASH does not measure a specific joint but the function of both upper extremities as a whole. With a score up to 29, patients usually no longer consider their upper limb disorder a problem. EQ visual analogue scale (EQ-5D-5 L VAS) was obtained and the EQ-5D-5 L-Index calculated [14].

### MR Imaging and analysis

Imaging was performed on a 1.5T MRI scanner (Ingenia; Philips Healthcare, Best, the Netherlands) with a shoulder coil and Philips MRI product software (Relase 5.7) between 07/08/2021 and 12/10/2021. For all six MRI examinations, T2-weighted (T2w) images were performed in three planes: axial, sagittal and coronal. Sagittal turbo-spin-echo sequence (T2w TSE) with echo times (TE) 60 ms, repetition time (TR) 3500 ms, slice thickness 3.3 mm. In axial and coronal imaging, metal artifact reduction sequence (MARS) technique was used to reduce the size and intensity of postoperative susceptibility artifacts from the orthopedic implants: T2w TSE MARS, TE 90 ms TR 4443 ms, slice thickness 3.3 mm. In 3 of the 6 examinations additional axial short inversion time inversion recovery (STIR) MARS sequences were performed after initial evaluation at the MRI scanner by one of the two radiologist readers during the examination to exclude fluid signal within the deltoid tendon indicating tendon tears: STIR MARS, TE 55 ms TR 3501 ms, slice thickness 4.3 mm.

**Fig. 2:**
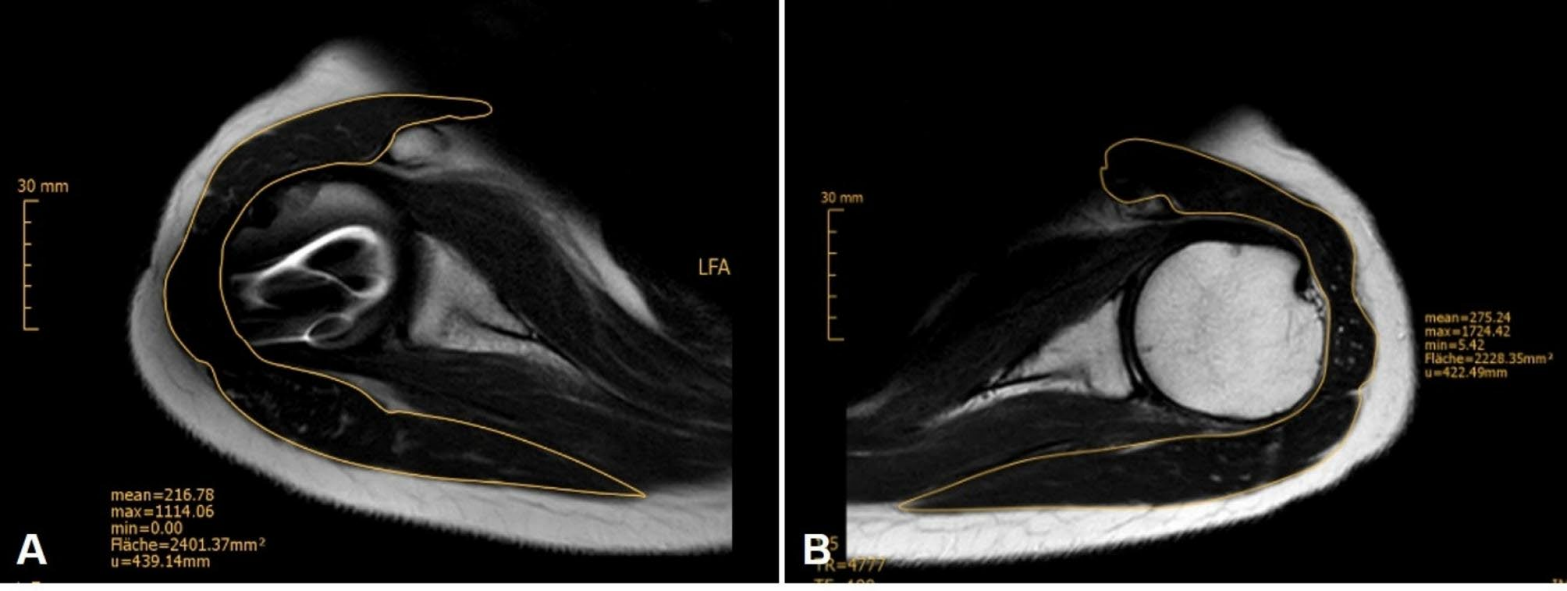
MRI analysis of an operated right humerus (**A**) and the contralateral side (**B**) of the same patient as shown in Fig. 1. The total area of the deltoid muscle (anterior, medial and posterior segments) was measured at the level of the humeral head center in the axial plane

**Fig. 3:**
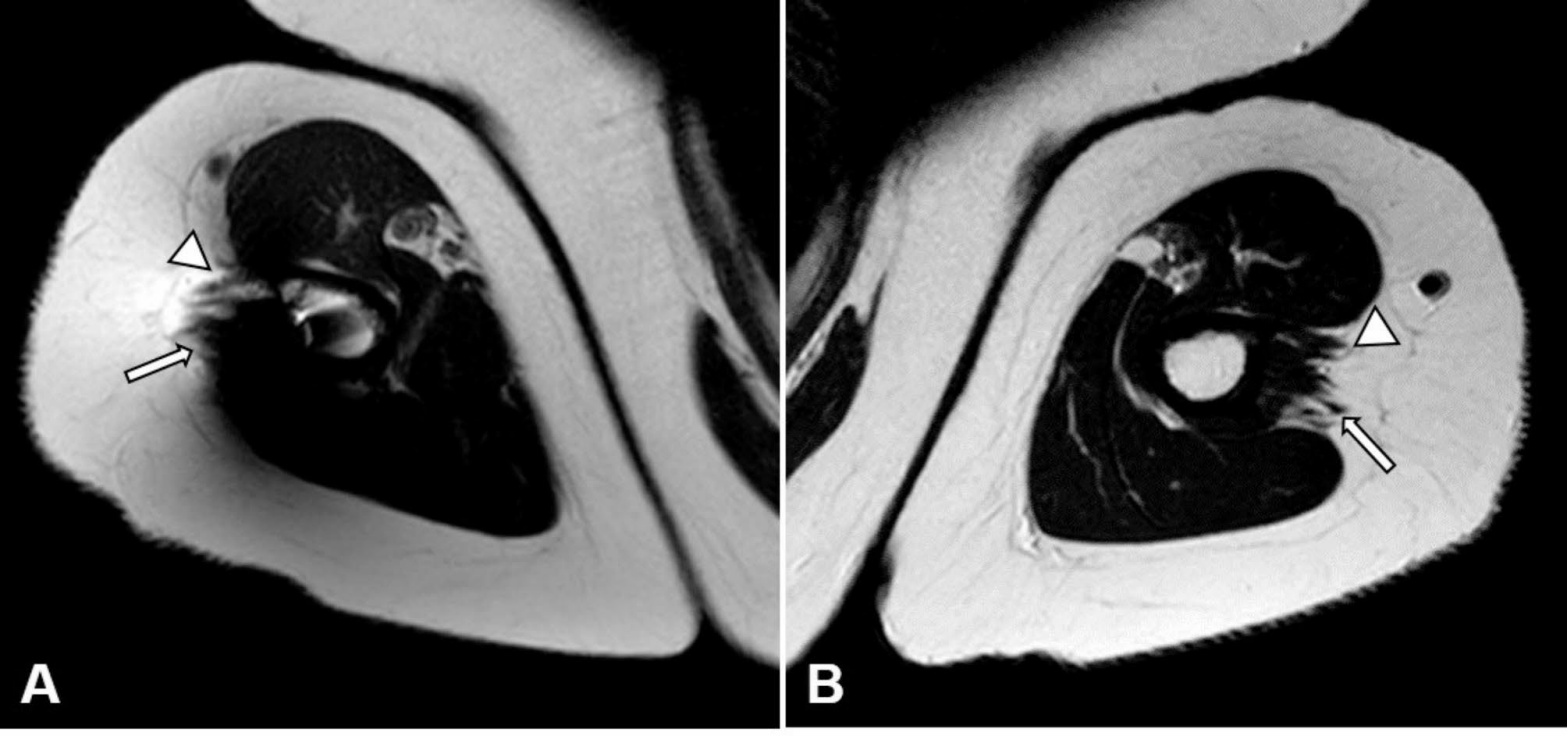
MRI analysis of an operated right humerus (**A**) and the contralateral side (**B**) of the same patient as shown in Figs. 1 and 2. The distal deltoid insertion with an intact anterior (triangles) and posterior (arrows) part is demonstrated on these axial images.

All MR images were analyzed in consensus reading by two experienced musculoskeletal radiologists (S.R. and C.M.), who each have 12 years of experience in musculoskeletal radiology. The following MR findings were evaluated for both shoulders of each patient. At the level of the humeral head center the total area of the deltoid muscle (anterior, medial and posterior segments, Fig. 2)) was measured in the axial plane by using an area calculation tool of the Picture Archiving and Communication System (Merlin PACS; Phoenix-PACS GmbH, Freiburg, Germany). The quality of the deltoid insertion, in particular the integrity of the anterior and posterior aspects of the insertion (Fig. 3), were analyzed. The rotator cuff was assessed to exclude a potential bias of the functional tests. Lesions of the rotator cuff were assessed by using a modification of the semi quantitative grading system of Zlatkin et al. [15] ranging from grade 1 (normal) to grade 4 (full thickness tear). The amount of fat infiltration was assessed of by using the classification system of Goutallier et al., modified by Fuchs et al. for MRI, which is based on the amount of fat relative to the amount of muscle ranging from grade 0 (no fat) to grade 4 (more fat than muscle) [16, 17]. Furthermore, major co-findings such as arthritis, labrum tear, cartilage lesions, or glenoid retroversion were evaluated.

## Results

Sixteen patients underwent MILPOH for metadiaphyseal fractures of the humerus between 03/2017 and 08/2020. Seven patients fulfilled all inclusion criteria and agreed to participate in the study. However, 1 patient had to be excluded since her MRI had to be aborted due to claustrophobia. Thus, complete data acquisition was obtained in 5 female and 1 male patient with a medium age of 63 years (range 52–69 years) between December 2020 and August 2021. Median follow-up was 29 months (range 12–48 months). The dominant side was affected in 2 patients. According to the AO-classification [18], the fracture patterns presented as follows: 3 × 12B2(i), 2 × 12B3(i), 1 × 12C3(i).

### Functional measurements and PROMS

**Fig. 4:**
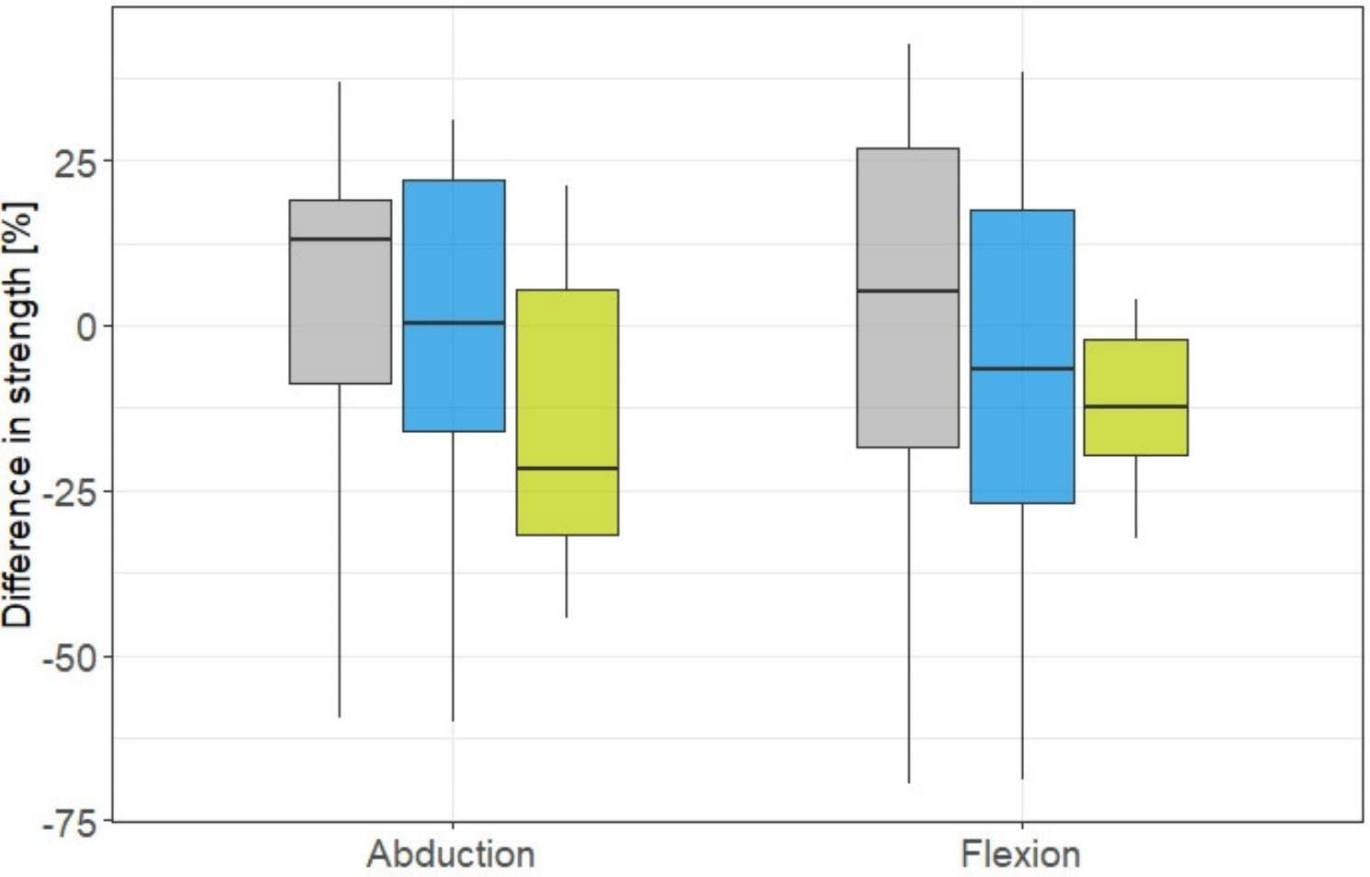
Shoulder function of the injured vs. uninjured side. Differences in strength for 30° (grey), 60° (blue), and 90° (green) of abduction and flexion, compared to the contralateral side. A positive value means better strength for the operated extremity. The box plot shows the median value (bold horizontal mark), the 25th-75th percentile (box) and the range (whiskers)

Median difference of abduction strength after MILPOH was + 13% for 30°, 0% for 60° and − 22% for 90° compared to the uninjured side. For flexion, the difference to the uninjured side was measured + 5% for 30°, -7% for 60° and − 12% for 90°. (Fig. 4).

Median CMS was 75 (66–82) for the operated extremity compared to 82 (77–90) for the uninjured side. Median age- and gender-adapted CMS was 88 (range 79–99) for the operated arm compared to 96 (range 89–107) for the contralateral extremity. Median OSS was 47, ranging from 40 to 48. Median DASH was 26 (range 15–36). EQ-5D-5 L VAS ranged from 81 to 95 with a median of 90. EQ-5D-Index was 1 (1–1). The individual results for each patient are provided in Table 1.

**Table 1:** Patient characteristics and outcome measurements

Patient	P1		P2		P3		P4		P5		P6	
Gender (m/f)	f		f		m		f		f		f	
Age (years)	52		57		69		58		67		67	
AO/OTA fracture classification	B3(i)		B2(i)		C3(i)		C3(i)		B2(i)		B2(i)	
Dominant arm (yes/no)	no		yes		no		yes		no		no	
Follow-up (months)	46		48		42		13		16		12	
OSS	40		45		48		42		48		48	
DASH	36		33		26		15		25		26	
Equation 5D VAS	90		90		90		83		81		95	
Equation 5D Index	1		1		0.887		1		1		1	
	injured	control	injured	control	injured	control	injured	control	injured	control	injured	control
CMS	73	83	66	78	79	82	74	90	75	77	82	82
CMS (gender- and age adapted [7]	87	99	79	93	86	89	88	107	90	93	99	99
Abduction strength at 30° (kg)	4.1	4.8	1.5	3.7	7.2	5.8	17.6	16.1	6.2	5.1	19.2	12.1
Abduction strength at 60° (kg)	3.0	3.4	1.4	3.5	6.4	4.4	5.2	6.3	7.1	5.3	17.1	15.0
Abduction strength at 90° (kg)	2.0	3.0	1.5	2.7	4.2	5.0	5.3	7.3	3.8	3.0	5.5	4.8
Flexion strength at 30° (kg)	4.2	5.2	1.4	4.6	5.3	6.3	12.9	16.3	6.3	4.6	15.0	8.6
Flexion strength at 60° (kg)	3.5	4.4	1.4	4.5	6.5	4.0	8.3	11.7	6.7	5.3	15.6	14.5
Flexion strength at 90° (kg)	2.3	2.9	2.1	2.5	5.0	4.8	4.1	4.5	3.9	3.9	5.0	7.4
Deltoid muscle area (mm2)	1951	2317	2335	2170	4665	3054	2401	2228	2452	2559	2378	3001
Deltoid muscle area (%, injured vs. control)	-16		8		53		8		-4		-21	

### MR imaging and analysis

Deltoid muscle area showed a heterogeneous pattern with a median difference of 2% and a wide range (-21% to + 53%) compared to the contralateral side, but no relevant fatty infiltration of the muscle was observed in any patient. The anterior and posterior parts of the distal deltoid insertion were intact in all patients as the central part of the insertion was exclusively penetrated by the plate. No relevant irregularities of the rotator cuff were found. In 2 cases, a partial tear < 50% (Zlatkin 3) of the infraspinatus tendon was diagnosed on the injured side. The subscapularis tendon demonstrated some minor lesions (Zlatkin 2–3) on both sides in the same patients.

## Discussion

It was the aim of this study to evaluate the implications of MILPO on the integrity of the deltoid insertion, muscle morphology, and functional outcome. MILPOH was associated with good functional and subjective outcome despite a minor reduction of abduction strength with increasing angles of abduction. Blunt advancement of the plate through the deltoid insertion exclusively affected the weaker central part of the distal deltoid insertion, leaving the strong anterior and posterior parts of the insertion intact in all patients. MILPOH did not affect the structural quality of the deltoid muscle.

### The distal deltoid insertion

The deltoid insertion covers a considerable area on the humeral bone. According to Rispoli et al., the width of the superior border of the insertion is 21.9 mm and 13.1 mm distally [19]. The length of the anterior insertion was measured 70.0 mm and 63.4 mm posteriorly. According to anatomical studies by Sakoma et al. [8], seven intramuscular tendons divide the deltoid muscle into corresponding anatomical and functional sections. The strong anterior and posterior insertion is formed by three intramuscular tendons each, with a considerable weaker intramuscular tendon in the middle. These strong anterior and posterior insertions form a V-like shape. When the plate is bluntly pushed distally, the plate is guided by these structures through the weaker central part of the insertion leaving the anterior and posterior insertions intact as previously shown in a cadaveric study and now confirmed by MR imaging in the current study [7]. The affected portions of the deltoid muscle corresponded with the proximal insertion arising from the lateral edge of the acromion leaving the clavicular- and most anterior portion of the acromion and the spinal portions undamaged. Strong interconnections of the deltoid tendon and fascia with the lateral intermuscular septum and the brachialis have been described [19]. These interconnections may be responsible for the preservation of the deltoid function despite partial detachment of the central intramuscular tendons. This view may be supported by the observation that neither muscular atrophy nor fatty infiltration of any part of the deltoid muscle was evident in our MR studies.

### Function and strength measurements of the deltoid muscle

We found a reduction in abduction strength, which was more distinct with increasing angles. Of interest, individual results showed a considerable variation and a general reduction of abduction strength was not a homogeneous finding. Some patients presented with even stronger strength compared to the contralateral side. This observation may indicate that a systematic and functional relevant damage to the distal deltoid insertion is not evident when the plate is pushed through the central portion of the distal deltoid insertion and that other factors may contribute to reduced abduction strength.

Isolated clinical testing of the deltoid muscle is difficult because no function is exclusively performed by the deltoid muscle alone. The deltoid muscle and the merging rotator cuff muscles all contribute to abduction and flexion of the shoulder. Data from the literature regarding the contribution of the deltoid and the rotator cuff muscles are quite controversial. Similar force distribution over the whole range of motion was found by some investigators while others described a position dependent force distribution [9, 20, 21]. In a recent study, Hecker et al. measured strength before and after performing an axillary nerve block [9]. They observed that the contribution of the deltoid muscle to abduction strength ranges from 24% at 0° to 75% at 120° of abduction in a linear manner. Flexion strength was 11% at 0°, and linearly increased to 70% for 120° of flexion. These results confirmed previous findings that the middle portion is not only the anatomically largest part of the deltoid muscle but also most important for shoulder abduction over the whole range of motion [9]. As the deltoid split approach affects exactly this portion of the muscle, impaired postoperative abduction strength may also be attributed to this approach. This view may be supported by a recent prospective randomized study, which demonstrated better functional outcome for the deltopectoral approach compared to the deltoid split for proximal humeral fracture fixation [22]. However, this finding is not uniform, other authors observed similar results for both approaches [23, 24].

### Functional outcome

Several clinical studies confirm a favourable outcome after MILPOH similar to our outcome measures [4, 5, 25]. However, none of these studies provided either general information regarding muscle strength or specific information of deltoid muscle function. Helical plates may also be used for fracture fixation. They are navigated anterior to the deltoid insertion without detaching it [6]. Functional outcome including CMS was similar to our results.

### Limitations

The current study is associated with considerable limitations. The study group is too small for a statistical analysis and there was no pre trauma data available from any patient. The deltoid is an important muscle for shoulder abduction and flexion, but the synergistic contribution of other muscles impairs an isolated measurement of the deltoid function within the current setting. One could hypothesize that decreased muscle strength and reduced deltoid muscle mass were not only influenced by injury and surgery, but handedness, activity level and compliance to the postoperative rehabilitation protocol as well. The dominant side was injured in only 2 out of 6 patients. Thus, the dominant side served as control in the majority of our patients. This fact may falsely increase the functional impairment in our study group after surgery. To exclude this potential bias, a much larger patient cohort is required.

The deltoid split approach may also be responsible for impaired abduction strength after surgery since the approach affects the integrity of the deltoid muscle. Furthermore, flexion strength might be reduced when branches of the axillary nerve are injured at the distal end of the deltoid split approach paralysing the anterior part of the deltoid muscle. Subacromial impingement caused by a high plate position on the greater tuberosity may also compromise function unrelated to injuries to the deltoid muscle or its insertion.

## Conclusions

MILPOH was associated with good functional and subjective outcome. Minor impairment of abduction strength was observed with increasing abduction angles. The reason for this impairment is unclear since MILPOH did not affect the structural quality of the deltoid muscle and the integrity of the strong anterior and posterior parts of its insertion remained intact. Due to the small size of the study group with the lack of a statistical analysis, this study may be considered preliminary research and caution should be taken when interpreting the herein presented results.

## Data Availability

The complete datasets used and/or analysed during the current study are available from the corresponding author on reasonable request.

## Abbreviations

MILPOH: Minimally invasive lateral plate osteosynthesis of the humerus
CMS: Constant-Murley Score
OSS: Oxford Shoulder Score
DASH: Disability of the Arm, Shoulder and Hand questionnaire
EQ-5D-5 L: VAS EQ visual analogue scale
MRI: Magnetic resonance imaging
MIPO: Minimally invasive plate osteosynthesis of the humerus
PHILOS: Proximal Humeral Internal Locking System
PROMS: Patient-reported outcome measures
MARS: Metal artifact reduction sequence
STIR: Short inversion time inversion recovery
